# Treatment of President Garfield

**Published:** 1881-11

**Authors:** T. Dwight Stow

**Affiliations:** Fall River, Mass.


### TREATMENT OF PRESIDENT GARFIELD.

T. Dwight Stow, M. D., Fall River, Mass.

[The Central New York Homœopathic Medical Society held its Fall meeting in Syracuse, September 15. The Society directed the Secretary to publish its proceedings in The Homœopathic Physician.

The Secretary had requested T. Dwight Stow, M. D., a member of the Society, and now resident in Fall River, Mass., to favor the Society with a paper on President Garfield's case. Dr. Stow sent a communication. It was read. On motion, a vote of thanks was tendered to Dr. Stow for his interesting paper; and the Secretary was instructed to select portions of it for publication.—Sec'y.]

Dr. Stow said: You, of course, see how difficult it is to criticise the treatment of the President's case, at this distance from the patient, and on the basis of "bulletins," and even the best correspondence; particularly, when we know *how little* the *attending surgeons know* of the extent and direction of the wound; the location of the bullet; the possible complications arising from its presence, such as pus pockets, sinuses communicating with the abdominal cavity, etc., etc. Yet, after the lapse of fifty-eight days, not only are the President's surgeons ignorant of the full nature of the wound, but of the *causes* of his greatly protracted illness; of the presence of pyæmia or of septicæmia; of the nature of the parotid enlargement; and—what seems very strange—their *inability* to harmoniously and clearly prognosticate! Making due allowance for human errors and shortsightedness, *it seems very strange* that they evidently know so little of his case; have neglected so much that reason dictates as of great importance; and have done so much not only uncalled for, but in its very nature *calculated to diminish the chances of recovery!* I would not be unreasonable, nor make groundless charges; but it does seem to me, this case, made so conspicuous by the official position of the patient, reveals such an amount of carelessness, disregard of physiological law, and ignorance of pathology, as to excite amazement and disgust! At the risk of tediousness or repetition, let us repeat the mistakes and errors:

1st. Twenty-one days elapsed, before the surgeons in charge discovered that the wound was mainly muscular!

2nd. Twenty-one days passed before they ascertained the fracture of the rib!

3d. Regardless of the lessons of history and of nature's laws, the President has been systematically and persistently drugged into unconsciousness, inanition and extreme debility!

4th. By their own confessions the surgeons have not been agreed as to the cause of the President's inability to digest food; as to the existence of pyæmia or septicæmia; nor as to the true nature of the parotid enlargement.

5th. At first, they ascribed the intractable character of the case to the wounding of the liver or other viscus; then to chronic dyspepsia; then to malaria; then to pyæmia; then to septicæmia, with nostalgia thrown in.

6th. When the parotid gland enlarged, they used discutients to systematize that which physiological law was localizing; thus retarding suppuration and the elimination of some irritating, perhaps septicæmic, quality. *It will be very interesting to know how much the interference with the enlargement of this gland had to do with the very unfavorable symptoms of Friday and Saturday, August 26th and 27th!*

7th. Since last Saturday they have stimulated the President with enemata of whiskey, better and more food; and it is very proper to ask the question, how long it will be before the President will have another relapse? Time will be well expended, if we further ask, and consider the following questions:

1st. Can we expect anything better of allopathic treatment?

2nd. If the old-school of medicine of to-day is not particularly better than it was in the days of "the sage," Paul of Ægina, centuries upon centuries ago; and if it learns nothing from failure and disaster, what *can* we hope of it, in the near or remote future?

3d. And, as to the President's case, what shall we think of *all* old-school boasts in the light of the ridiculous failures of diagnosis; of treatment; and of prognosis? Whither went the bullet? Is it in the psoas muscle? or, in the quadratus; or in the iliac fossa; or in the gluteal region? Or is it, as Dr. Bliss suggests, in the infra pelvic perineal region, ulcerating its way through and into the rectum? And if so, whence goes much of the pus that must escape the lower or inner opening of the "twelve and one-half inches of channel?" *What a howl* would have gone up from Maine to Texas, and from Cape-Cod to Cape Prince of Wales, had half the blunders already witnessed occurred under homœopathic treatment of the President's case!

Taking Drs. Agnew, Hamilton, and the rest, as authority for the statement "that the wound was muscular, and by no means a fatal one," and the President *now* a powerless, almost voiceless and insensible person, "whose joints are his most prominent parts;" when "fair science, and art," and friends, and money, have been invoked; failure! failure!! failure!!! is stamped *as with a branding iron*, upon everything but the President's endurance! Whether the President lives or dies, old-school medication will receive a *staggering blow*, and all the more so because the patient is a conspicuous one.

Let it be *distinctly understood*, that in criticising the management of the President we must admit the possibility of the lodgment of the bullet, in some *deep recess*, as in the crest of the ilium; or in the lower third of the psoas; or in the iliac fossa; and that, by its own weight, and the irritation its presence produces, the sinus may be deepened, pus chambers formed, and much tedious suppuration follow. But, this will not materially affect the charge we make, that the drugging and tinkering resorted to have been such as to seriously impair all his functions; to produce glandular obstruction; to invite inanition and exhausting emaciation; and to place the patient at death's door! If the treatment of the President be a fair illustration of the *ways*, *means* and *methods*, of so-called *regular* medicine, *Heaven deliver us!*

Up to August 13th, the President was given eight hundred grains of quinine, and not far from four hundred grains of opium (*New York Herald* of August 13). Besides these, he has had Tokay wine, sub-nitrate of bismuth, etc. Too much has been done.

Why should a superficial gun-shot wound, attended with so little hemorrhage, and not worse suppuration, cause such marked debility and emaciation, and bring the patient so near death's door? The true answer is not to be found in the presence of, or injury produced by, Guiteau's bullet, if Dr. Agnew's statements as to the direciion, course, and location of the bullet be true. No, we must go farther in our search. We must refer to the condition of the President's nervous system, and from that to his digestive system. We are to look for the solution of the problem to the action of stimulants, on the one hand, and, on the other, to the more pernicious effects of opium or some of its alkaloids. Quinine and opium (morphia) exert a direct and very powerful influence upon the nervous centres and spinal ganglionic system, often followed by, not only temporary, but permanent, functional and structural changes, such as congestion, anæmia, perverted function, and impeded and paralyzed innervation. The President grows weak and poor, and sinks, because nutrition has been impaired and suspended; not because he has lost so much by the irritation of the bullet, or by suppuration. Upon false premises has rested pernicious medication. Neglect to ascertain the route of the bullet, and, if possible, to extract it, led to the conclusion that the liver and peritoneum, or other viscera, were wounded; and consequent starvation, loss of time, and unnecessary suppuration followed. After twenty-one days, Dr. Agnew found, and the rest coincided, that the bullet penetrated the "quadratus," and perhaps the "psoas" muscle, not the liver or any noble organ, and that the wound was simple and not dangerous. Under any morbid condition, the vital powers and vital processes should be husbanded and allowed full and free play; for upon such freedom the reparation of tissue and the maintenance of physical integrity depend.

Not in that light have President Garfield's counsel acted. We do not impute to them wrong intentions; far from it. But, instead of being guided by natural laws, they have attempted to make nature conform to their ideas. When nature was resting, or getting ready for work, they have stimulated. When assimilation, secretion and excretion should have been active and untrammeled, they have impeded and paralyzed innervation. Eight hundred grains of quinine! Four hundred grains of opium! No wonder the President sinks nigh unto death! God only knows. To human eyes the President seems doomed! If he dies, not alone Guiteau's bullet, but unscientific and disastrous medication will have done the work! Old-School authorities, such as Hufeland, Christison, Pareira Dunglison, Wood and Bache in the U. S. Dispensatory, and other writers, have described the pernicious effects of such heroic drugs.

The state of narcotism and insensibility in which the President has been kept (and they have mistaken it for sleep!) *has been a stumbling block and delusion all along;* but *emphatically* at the time when the President needed his *full senses* that he might *detect, describe,* and *locate pain,* beating, throbbing, and other diagnostic phenomena! The principle involved here is of wider significance than the advocates of palliation may know of, or be willing to admit. The use of narcotics, besides blinding diagnosis and prognosis, has a disastrous, and ofttimes *fatal,* effect!

Friday, September 9th, a bulletin informed us that Dr. Hamilton had found the bullet in the right iliac fossa, and under the iliac artery. Good! Now what? We shall see. At this writing (it is September 12,) there comes just at this moment the startling news that the President is much worse! Probably our worst fears are to be realized!

[A portion of Dr. Stow's paper contained a differential diagnosis of the location of the bullet. The autopsy having disclosed the course and situation of the bullet, Dr. Stow was asked whether his diagnosis should be published. His reply is the following.—SEC'Y.]

I do not think it worth while to publish the "diagnostic" sheets of my late article. There was but one theory, advanced in that part of the paper, that came any where near the truth as revealed by the autopsy; and that was the lodgment of the bullet in or near the vertebral column; that was marked C, or D. Of course, in the commencement of the article, I stated the great difficulty of making a correct diagnosis at this distance, and upon the strength of official bulletins and newspaper correspondence. Then too, I concluded that Guiteau fired upon the President at nearly point-blank range, but not quite that. He must have fired upon the President at an angle of 45°, or even less. In all my theories I kept in mind Dr. Agnew's statement at the time he was first called, and when he found the fracture of the rib; also the manner in which he opened the wound, viz. downward.

You will recollect he said the ball penetrated "the quadratus, or psoas and might be in the crest of ilium."

Another statement, made by Dr. Bliss, was "the depth of the wound or sinus was eighteen inches!" This was made so many times that I was made to think that the obscurity of the locality of the bullet and the position of the assassin warranted the conclusion that the bullet lay in the body of the psoas, or in the iliac fossa. But for the abscess found in the region of the gall-bladder (an abscess cavity) the *wound* should not be regarded as necessarily fatal. Of course, the perforation of the vertebra was *very serious* and greatly increased the danger; but very bad cases of idiopathic lumbar abscess recover nicely, and many traumatic diseases of the spine recover. I have had bad cases of lumbar, psoas, and perityphlitic abscess, and do not recollect having lost one. Other physicians can say more. What I said of the treatment of the President's case, I wish to repeat; viz.

First. Neglect to make a better or clear diagnosis in the President's case increased the danger, regardless of the true locality of the bullet. *For*, from the mouth of wound to the point *at* which the bullet *emerged* from the last dorsal vertebra, and became encysted in the mesentery behind the pancreas, was not more than seven inches, and the wound was *straight.* Careful probing would have given the direction and depth of wound; A well-adjusted gold drainage tube (flexible) and a better position in bed for the easy and free discharge of disintegrating bone and suppurating soft tissues, would have immensely aided the patient and surgeons. The autopsy showed the bullet to have been completely encysted.

Second. It is possible that the 4x6 abscess in the hepatico-colic region, was caused by pressure or puncture of the serous investment of the liver, by a spicula of broken rib. Had this been ascertained at once, as it should have been, who can say that this abscess would have followed?

Third. What caused secondary hemorrhage from the mesenteric artery? Undoubtedly, the friability of that artery. But why did the artery become friable? Because nutrition and the various processes upon which the repair of torn or otherwise injured tissues depends, was seriously disturbed, and nothing so seriously disturbs the harmonious and normal action of the organism, as quinine, opium, whiskey, and so on.

The autopsy shows the desperate character of the case; but the worse the case, the greater should be the care used. Was proper care used? Evidently not, for proper diagnosis, proper knowledge, proper treatment were *not* used.

The first signs of failure were of digestion; then of assimilation; then of reparation of tissue; then of lymphatic and parotid disturbance; then of pyæmia or septicæmia. I cannot throw off the impression that the President's only chances were ruthlessly sacrificed. Time will probably give us the *true* solution of this case.

